# The Crimean-Congo Hemorrhagic Fever Virus NSm Protein Is Dispensable for Growth In Vitro and Disease in Ifnar^-/-^ Mice

**DOI:** 10.3390/microorganisms8050775

**Published:** 2020-05-21

**Authors:** Stephen R. Welch, Florine E. M. Scholte, Jessica R. Spengler, Jana M. Ritter, JoAnn D. Coleman-McCray, Jessica R. Harmon, Stuart T. Nichol, Sherif R. Zaki, Christina F. Spiropoulou, Eric Bergeron

**Affiliations:** 1 Viral Special Pathogens Branch, Division of High-Consequence Pathogens and Pathology, Centers for Disease Control and Prevention, Atlanta, GA 30333, USA; yos6@cdc.gov (S.R.W.); kyj7@cdc.gov (F.E.M.S.); jspengler@cdc.gov (J.R.S.); flj7@cdc.gov (J.D.C.-M.); vrx7@cdc.gov (J.R.H.); stn1@cdc.gov (S.T.N.); ccs8@cdc.gov (C.F.S.); 2 Infectious Diseases Pathology Branch, Division of High-Consequence Pathogens and Pathology, Centers for Disease Control and Prevention, Atlanta, GA 30333, USA; vtr0@cdc.gov (J.M.R.); sxz1@cdc.gov (S.R.Z.)

**Keywords:** CCHF, Crimean-Congo hemorrhagic fever, reporter virus, fluorescent protein reporter, Ifnar^-/-^, Ifnar knockout mouse, in vivo imaging, non-structural protein, NSm, ZsGreen1, reverse genetics

## Abstract

Crimean-Congo hemorrhagic fever virus (CCHFV) is a tri-segmented, tick-borne nairovirus that causes disease of ranging severity in humans. The CCHFV M segment encodes a complex glycoprotein precursor (GPC) that undergoes extensive endoproteolytic cleavage, giving rise to two structural proteins (Gn and Gc) required for virus attachment and entry, and to multiple non-structural proteins (NSm, GP160, GP85, and GP38). The functions of these non-structural proteins remain largely unclear. Here, we investigate the role of NSm during infection by generating a recombinant CCHFV lacking the complete NSm domain (10200∆NSm) and observing CCHFV ∆NSm replication in cell lines and pathogenicity in Ifnar^-/-^ mice. Our data demonstrate that the NSm domain is dispensable for viral replication in vitro, and, despite the delayed onset of clinical signs, CCHFV lacking this domain caused severe or lethal disease in infected mice.

## 1. Introduction

Crimean-Congo hemorrhagic fever (CCHF) is a zoonotic disease caused by Crimean-Congo hemorrhagic fever virus (CCHFV; order, *Bunyavirales*; family, *Nairoviridae*; genus, *Orthonairovirus*) [1]. CCHFV is endemic in southeastern Europe, Africa, the Middle East, and Asia, correlating with the geographic distribution of CCHFV’s primary vector and reservoir, *Hyalomma* spp. ticks [2]. Human infections occur via tick bites, nosocomial transmission, or direct contact with the blood or tissues of infected animals [3]. Human disease generally manifests as a sudden-onset, non-specific febrile illness that progresses in severe cases to petechial rash, ecchymoses, and other hemorrhagic manifestations, with case fatality rates of 5%–30% [4].

CCHFV possesses a negative-sense, single-stranded RNA genome composed of three segments termed large (L), medium (M), and small (S). Each segment contains at least one open reading frame. The L segment encodes the RNA-dependent RNA polymerase, and the S segment encodes the nucleoprotein (NP) and a non-structural protein (NSs) thought to play a role in regulating apoptosis in infected cells [5]. The M segment encodes a glycoprotein precursor (GPC) that is post-translationally cleaved by cellular proteases into the structural envelope glycoproteins Gn and Gc; the non-structural proteins NSm, GP160, GP85, and GP38; and two uncharacterized putative proteins, GPmuc and ProGc [6]. While Gn and Gc are required for the attachment and entry of viral particles [6], the functions of the non-structural proteins remain unclear.

M segment-encoded non-structural proteins (NSm) are found in multiple members of the order *Bunyavirales*, including viruses classified in the *Nairoviridae*, *Peribunyaviridae*, *Phenuiviridae*, and *Tospoviridae* families, although no significant NSm sequence homology is observed between families [7]. Various studies have attempted to elucidate the role of these NSm proteins using reverse genetics systems, finding NSm to be non-essential for in vitro replication and assembly in Maguri virus [8], Oropouche virus [9], Rift Valley fever virus (RVFV) [10,11], and Schmallenberg virus (SBV) [12]. In contrast, NSm was shown to be essential for replication of Bunyamwera virus and has further been identified as a scaffold protein involved in virus assembly and morphogenesis, although attempts at complete deletion of NSm have not been reported [13,14]. No studies have investigated the potential role of nairovirus NSm proteins. The NSm domain is not universally found in nairovirus genomes; NSm orthologs are only found in nairoviruses that phylogenetically cluster with CCHFV, including Nairobi sheep disease virus, Dugbe virus, Kupe virus, and Hazara virus. Until recently, investigating the role of nairovirus NSm proteins has been restricted by the absence of reverse genetics systems [15,16].

Here, we investigate the role of CCHFV NSm by generating a recombinant virus lacking the NSm protein (10200∆NSm) and a variant expressing a ZsG reporter protein (10200∆NSm/ZsG). Both ∆NSm viruses could be propagated in multiple cell lines, and 10200∆NSm/ZsG caused lethal disease in type I interferon receptor knockout mice (Ifnar^-/-^) [17]. These results highlight the non-essential role of CCHFV NSm for viral growth in mammalian cells and for pathogenicity in an interferon-compromised CCHF mouse model.

## 2. Materials and Methods

### 2.1. Biosafety and Ethics Statement

All CCHFV infections and rescue attempts were performed in biosafety level 4 (BSL-4) facilities at the Centers for Disease Control and Prevention (CDC; Atlanta, GA, USA). Experiments involving cDNA encoding viral sequences were performed in accordance with approved Institutional Biosafety Committee protocols. All animal procedures were approved by the CDC Institutional Animal Care and Use Committee (#2797SPEMOUC) and conducted in accordance with the Guide for the Care and Use of Laboratory Animals [18]. The CDC is fully accredited by the Association for Assessment and Accreditation of Laboratory Animal Care International. Procedures conducted with CCHFV-infected animals were performed in the CDC BSL-4 laboratory.

### 2.2. Cells

BSR-T7/5 (a kind gift from K.K. Conzelmann, Ludwig-Maximilians-Universität, Munich, Germany), A549, and Huh7 cells were cultured in Dulbecco’s Modified Eagle’s Medium (DMEM) supplemented with 5% (*v*/*v*) fetal calf serum, non-essential amino acids, 1 mM sodium pyruvate, 2 mM L-glutamine, 100 U/mL of penicillin, and 100 μg/mL of streptomycin.

### 2.3. Virus Rescue and Titration 

The generation of recombinant CCHFV based on strain IbAr10200 (10200), and a variant expressing a fluorescent reporter protein (10200/ZsG) have been described previously [15,19]. Recombinant 10200 ∆NSm viruses were generated by removing the NSm coding region (defined as the region between amino acids 841 and 995 in the GPC [20]) from the M genome segment reverse genetics plasmid. This modified M segment plasmid, in conjunction with the standard L genome segment and a plasmid containing either the standard S genome or a modified S genome segment encoding the ZsG ORF, was used to rescue the 10200∆NSm or the 10200∆NSm/ZsG reporter virus, respectively. Recombinant viruses were rescued by transfecting Huh7 cells with the required plasmids and harvesting supernatants 4–5 days post transfection. This P0 stock was then used to generate the working P1 stocks by infecting BSR-T7/5 cells and harvesting cell culture supernatants 2–3 days post infection (dpi). Viral stock titers were determined by tissue culture infective dose 50 (TCID_50_) assays in BSR-T7/5 cells using ZsG expression or immunostaining [21]. All virus stocks were determined to be free of mycoplasma, and genome sequences were confirmed using Illumina sequencing technology.

### 2.4. Western Blot Analysis

Cell monolayers were harvested in 2× Laemmli sample buffer (without reducing agent), denatured at 95 °C for 10 min (except samples probed with α-CCHFV Gc, which were heated at 50 °C for 10 min), separated by SDS-PAGE on 4%–12% bis-tris gels, and transferred via semi-dry blotting to nitrocellulose membranes. The primary antibodies used were against CCHFV nucleoprotein (NP; IBT Bioservices, #04-0011), CCHFV Gn (a kind gift from A. Mirazimi), CCHFV Gc (11E7, BEI Resources), ZsGreen1 (Clontech, #632598), and tubulin (Sigma-Aldrich, #T5168). After probing with HRP-linked secondary antibodies (ThermoFisher Scientific #32260 and #32230), the proteins were visualized using a Fast Western Blot kit with SuperSignal West Dura HRP substrate (ThermoFisher Scientific), and blots were imaged with a ChemiDoc MP system (Bio-Rad).

### 2.5. Mouse Infections

Female B6.129S2-Ifnar1tm1Agt/Mmjax mice (MMRRC 032045-JAX; 7–8 weeks of age) were inoculated subcutaneously in the inter-scapular region with 100 TCID_50_ of 10200∆NSm/ZsG diluted in DMEM (total volume, 100 μL), in parallel with previously reported studies of 10200 and 10200/ZsG [22]. Mice were housed in a climate-controlled laboratory with a 12 h day/12 h night cycle, provided with sterilized commercially available mouse chow and water ad libitum, and group-housed with sterile bedding in an isolator-caging system (Thoren Caging Systems, Inc.). Mice were humanely euthanized with isoflurane vapor at the indicated time points, or when clinical illness scores based on piloerection, neurological signs, changes in mentation, ataxia, dehydration, and/or dyspnea indicated that the animal was in distress or in the terminal stages of disease. A recorded weight loss of >20% from baseline (−1 dpi) also necessitated euthanasia when seen in conjunction with any other clinical sign of disease.

### 2.6. Fluorescent Microscopy and Imaging

ZsG fluorescence in infected cells was visualized and imaged using an EVOS Cell Imaging System (ThermoFisher Scientific). ZsG fluorescence was visualized and imaged in situ using a Canon PowerShot G12 camera in conjunction with a Dark Reader camera filter (#AF580), Dark Reader Spot Lamp (#SL 10S), Dark Reader Hand Lamp (#HL34T), and Dark Reader glasses (#AG16), all from Clare Chemical Research (Dolores, CO, USA).

### 2.7. Quantitative RT-PCR

RNA was extracted from whole blood (25 µL) and homogenized tissue samples (~1 mm^3^) using a MagMAX-96 Total RNA Isolation Kit (ThermoFisher Scientific) on a 96-well ABI MagMAX extraction platform with a DNase I treatment step according to manufacturer’s instructions. RNA was quantified using a one-step, real-time RT-PCR targeting NP (forward primer, ATGAACAGGTGGTTTGAAGAGTT; reverse primer, TGGCACTGGCCATCTGA; probe, 6FAM-TGTCCAAATTGGGAACACTCTCGCA-BBQ; Integrated DNA Technologies) and normalized to 18S RNA levels (ThermoFisher Scientific) with a SuperScript III Platinum One-Step qRT-PCR Kit (ThermoFisher Scientific) according to the manufacturer’s instructions. CCHFV S genome copy numbers were calculated using a standard curve of in vitro-transcribed RNA of known copy number.

### 2.8. Histology and Immunohistochemistry

Tissue specimens were fixed in 10% neutral buffered formalin and gamma-irradiated (2 × 10^6^ rad). Tissues were routinely processed for paraffin embedding, sectioning, and staining with hematoxylin and eosin. For the immunohistochemistry assays, slides were stained with rabbit α-CCHFV NP pAb (IBT Bioservices, #04-0011) diluted 1:1000, as previously described [23].

### 2.9. Graphing and Statistical Analyses

Survival statistics were calculated using the Mantel–Cox test. Weight statistics and PCR viral load data were calculated using multiple *t*-tests with the Holm–Sidak multiple comparison test. All analyses were performed and graphs generated using GraphPad Prism v8.0 software.

## 3. Results

### 3.1. Generation and In Vitro Characterization of Recombinant CCHFV Lacking the NSm Domain

**Figure 1:**
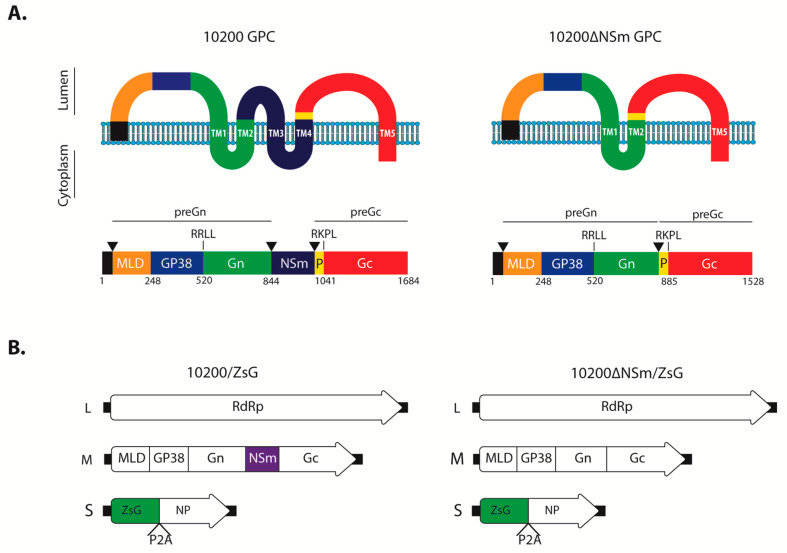
10200∆NSm/ZsG design. (**A**) Schematic showing the predicted membrane topology of the glycoprotein precursor (GPC) and ∆NSm mutant encoded by the recombinant M segments of either 10200/ZsG or 10200∆NSm/ZsG. TM, predicted transmembrane domain; P, ProGc; black triangles, predicted signal peptidase cleavage site; RKLL and RKPL, predicted S1P/SKI-1-like protease cleavage signals. Numbers indicate the first amino acid position of each domain. (**B**) Schematics of Crimean-Congo hemorrhagic fever virus (CCHFV) L (large), M (medium), and S (small) genome segments used to rescue the 10200/ZsG and 10200∆NSm/ZsG viruses. RdRp, RNA-dependent RNA polymerase; MLD, mucin-like domain; ZsG, ZsGreen1; NP, nucleoprotein; P2A, porcine teschovirus-1 2A self-cleaving peptide.

The CCHFV glycoprotein precursor (GPC) is co- and post-translationally processed by cellular proteases into multiple proteins, including the structural Gn and Gc, and the predicted double membrane-spanning non-structural NSm protein [20]. To investigate the importance of the NSm protein during CCHFV infection, we designed a construct that would maintain GPC topology based on three criteria: the predicted boundaries of NSm [20]; predicted GPC transmembrane domains; and alignment with Erve virus, a nairovirus that lacks NSm but shares significant homology with CCHFV GPC [24], including the bordering region of the second transmembrane domain (TM2) and ProGc (Figure 1a). Based on these criteria, the nucleotides encoding the P841–A995 region were removed from the M segment rescue plasmid. This strategy allowed the retention of the Gn TM2 signal peptidase cleavage site C-terminal of TM2 and the downstream preGc regions (ProGc to Gc C-terminus amino acids) to maintain correct post-translational cleavage, processing, and trafficking of both Gn and Gc. Using the established CCHFV reverse genetics system based on the prototypic IbAr10200 strain, we rescued a recombinant virus lacking the NSm domain, termed 10200∆NSm (Figure 1a). In addition, we generated a reporter version of 10200∆NSm expressing the green fluorescent protein ZsGreen1 (ZsG) as part of the S segment, as previously described for wild-type IbAr10200 [19] (Figure 1b).

**Figure 2:**
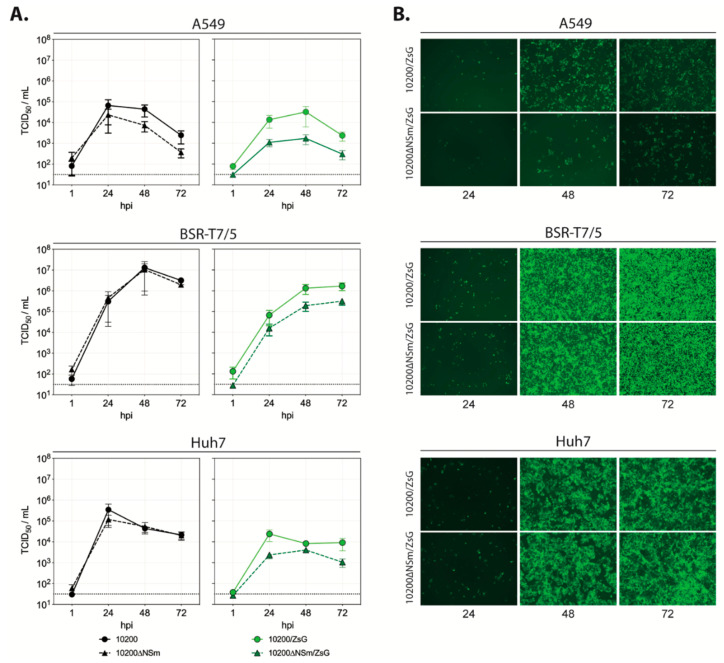
In vitro characterization of recombinant 10200 variants. (**A**) Growth kinetics of 10200 (black circle), 10200∆NSm (black triangle), 10200/ZsG (green circle), and 10200∆NSm/ZsG (green triangle) recombinant CCHF viruses in A549, BSR-T7/5, or Huh7 cells infected at a multiplicity of infection (MOI) of 0.1. Cells were incubated with virus inoculum for 1 h and washed once with PBS before fresh media was added. Titers (TCID_50_/mL) were determined at the indicated timepoints. Represented are the mean and standard deviation of triplicate experiments (each individual experiment n = 3). (**B**) A549, BSR-T7/5, or Huh7 cells infected with either 10200/ZsG or 10200∆NSm/ZsG at an MOI of 0.1 were imaged 24, 48, and 72 h post infection using fluorescent microscopy examining ZsG expression levels (images shown are at ×10 magnification).

To investigate the importance of NSm for in vitro viral replication, the growth kinetics of 10200∆NSm and 10200∆NSm/ZsG were compared to the kinetics of wild-type parental viruses (recombinant 10200 and 10200/ZsG) in human A549 lung epithelial, human Huh7 hepatoma, and hamster BSR-T7/5 fibroblast cell lines (Figure 2a). For all viruses, highest titers were observed in BSR-T7/5 cells and lowest titers in A549 cells. In BSR-T7/5 and Huh7 cells, the titers of 10200 and 10200∆NSm were similar at all time points, but in A549 cells, titers of 10200∆NSm were approximately 10-fold lower than the 10200 titers between 24 and 72 h post infection. In all cell lines, 10200∆NSm/ZsG grew less efficiently than either 10200∆NSm or 10200/ZsG, with an average 100- to 10-fold reduction in titers observed, respectively. Consistent with the titers, ZsG expression was reduced in 10200∆NSm/ZsG-infected A549 cells compared to that in 10200/ZsG-infected cells at all time points, while the progression and intensity of ZsG expression were comparable for both reporter viruses in BSR-T7/5 and Huh7 cells (Figure 2b). Western blot analysis confirmed that correct post-translational cleavage of Gn and Gc was maintained in the ∆NSm mutant (Figure S1).

### 3.2. Pathogenicity of 10200∆NSm/ZsG in Ifnar^-/-^ Mice

**Figure 3:**
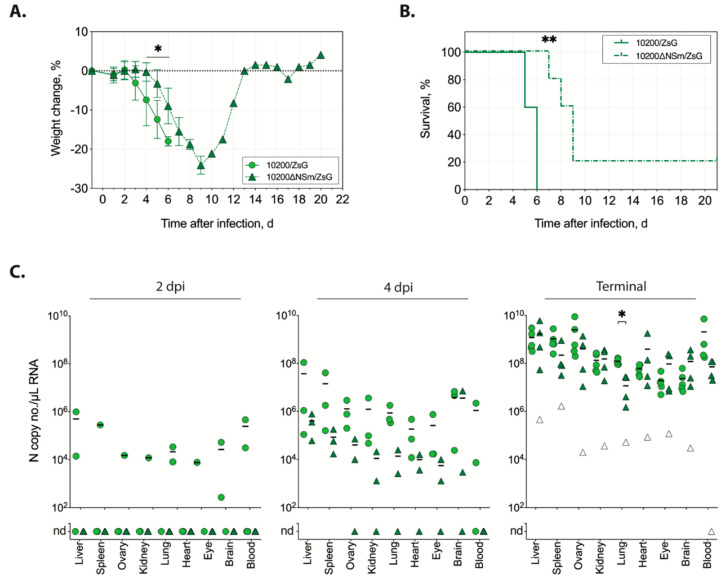
In vivo characterization of recombinant 10200 variants in the Ifnar^−/−^ mouse model. (**A**) Weight change and (**B**) survival of Ifnar^-/-^ mice inoculated subcutaneously with 100 TCID_50_ of 10200/ZsG (green circle and solid line; n = 5) or 10200∆NSm/ZsG (green triangle and dashed line; n = 5). Lines represent the mean weight change of all individuals on that day ± standard deviation (SD). Weight data were analyzed by multiple *t*-tests; survival statistics were calculated using the Mantel–Cox test; * *p* < 0.05; ** *p* < 0.01. (**C**) Viral RNA quantitation at 2 days post infection (dpi; n = 3 each), at 4 dpi (n = 3 each), when euthanasia criteria were met, or at study completion (21 dpi, n = 5 each). Individual animal data are represented for the 10200 N copy number/µL RNA in each tissue type from mice infected with either 10200/ZsG (circles) or 10200∆NSm/ZsG (triangles). The single surviving 10200∆NSm/ZsG-infected mouse in the terminal group is represented as a white triangle. Lines represent the mean of positive values. Nd, none detected. Statistics were calculated using multiple *t*-tests; * *p* < 0.05.

The replication and in vivo pathogenicity of recombinant CCHFV expressing ZsG are similar to those of wild-type virus [19,22]. Therefore, using the ZsG-expressing CCHFV variants, we compared replication and pathogenicity of virus with or without NSm expression in the Ifnar^-/-^ mouse model. The animals were infected subcutaneously with 100 TCID_50_ of either 10200/ZsG (n = 11) or 10200∆NSm/ZsG (n = 11). From each group, three animals were euthanized at 2 and 4 dpi to assess viral dissemination, and five animals were euthanized when end-point euthanasia criteria were reached (terminal group). Weight loss in 10200/ZsG-infected mice was consistent with previous reports of mice infected with wild-type recombinant 10200; it was observed from 2 dpi and progressed until animals succumbed at 5–6 dpi [17,22,25]. However, differences in weight loss were significant (*p* > 0.01) between 10200∆NSm/ZsG- and 10200/ZsG-infected mice beginning at 4 dpi, with slower weight loss observed in 10200∆NSm/ZsG-infected mice (Figure 3a). Survival was also significantly (*p* > 0.001) different in 10200∆NSm/ZsG-infected mice, although 80% (4 of 5) of the 10200∆NSm/ZsG-infected mice eventually met end-point criteria 7–9 dpi (Figure 3b). The single surviving 10200∆NSm/ZsG-infected mouse recovered from disease after demonstrating clinical signs similar to the mice that succumbed, including weight loss, hunched posture, ruffled fur, and decreased activity.

To compare dissemination and disease kinetics of 10200∆NSm/ZsG to 10200/ZsG, we examined several tissues for the presence of viral RNA (Figure 3c). At 2 dpi, low levels of viral RNA were widely detected in tissues of two out of three 10200/ZsG-infected mice, whereas no viral RNA could be detected in any of the 10200∆NSm/ZsG-infected mice. At 4 dpi, viral RNA was detected in all mice in both the 10200/ZsG- and 10200∆NSm/ZsG-infected groups; however, RNA levels were several logs lower in the 10200∆NSm/ZsG-infected mice. In terminal animals, viral RNA was also detected in all tissue samples, and high levels of RNAemia were detected in 100% of both 10200/ZsG-infected (5 of 5) and 10200∆NSm/ZsG-infected (4 of 4) mice. Overall, RNA levels of both viruses were highest in the liver and spleen. There was no significant difference in RNA levels in the terminal animals aside from those detected in the lungs of 10200∆NSm/ZsG animals compared to 10200/ZsG animals (*p* > 0.01). In the single surviving 10200∆NSm/ZsG-infected animal at study completion (21 dpi), RNA was detected in all tissues except the blood, although levels were, on average, 10,000-fold lower compared to those in animals that succumbed.

### 3.3. In Situ Detection of Fluorescence in Mice Infected with 10200/ZsG or 10200∆NSm/ZsG

**Figure 4:**
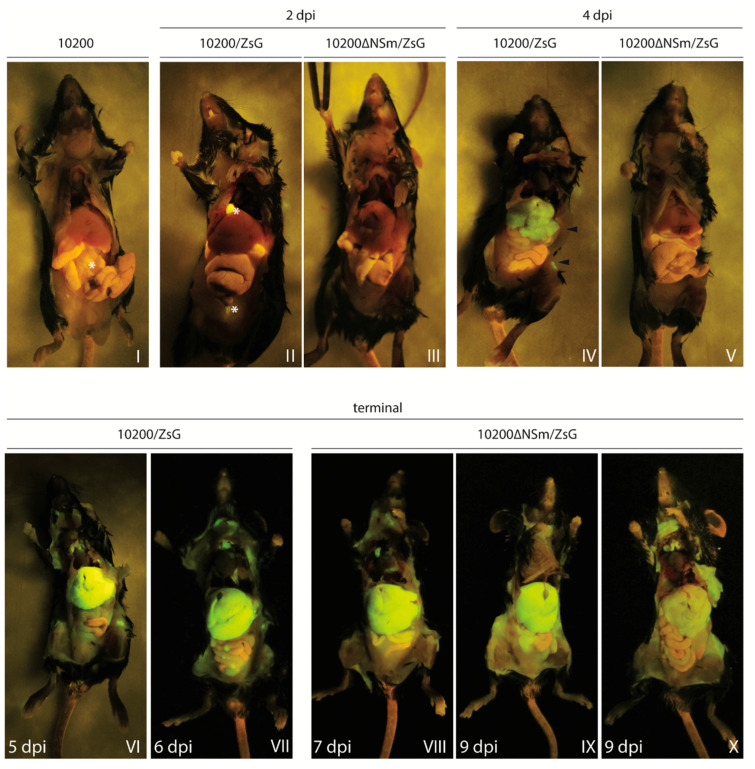
In situ detection of ZsG fluorescence in Ifnar^−/−^ mice infected with 10200, 10200/ZsG, or 10200∆NSm/ZsG. Ifnar^−/−^ mice were infected subcutaneously with 100 TCID_50_ of either 10200/ZsG (n = 11) or 10200∆NSm/ZsG (n = 11) and euthanized at 2 dpi (n = 3) or 4 dpi (n = 3) or allowed to progress until end-point euthanasia criteria were reached (terminal group, n = 5). A single 10200-infected mouse was also euthanized 2, 4, or 5 dpi to determine levels of autofluorescence and background fluorescence, with the 2 dpi animal represented in Panel I. In Panels I and II, the strong autofluorescence observed in the gall bladder and bladder, and slight autofluorescence in the intestinal tract are marked with *. At 2 dpi (Panels II and III), ZsG fluorescence was not detected in any tissue. At 4 dpi (Panels IV and V), ZsG fluorescence was detected in the livers and inguinal lymph nodes (both marked with arrowhead) of 10200/ZsG-infected mice (n = 3), but fluorescence was not observed in any of the 10200∆NSm/ZsG-infected mice (n = 3). In 10200/ZsG-infected terminal animals (Panels VI and VII), extensive ZsG fluorescence was observed in the liver and lymphoid tissues (brachial, mediastinal, inguinal, and lumbar lymph nodes) in animals euthanized at 5 or 6 dpi. Levels of fluorescence in the tissues were similar between animals at these terminal time points. In 10200∆NSm/ZsG-infected terminal animals (Panels VIII–X), fluorescence was detected in the same tissues as in 10200/ZsG-infected animals. Fluorescence intensity, however, was inconsistent between the 10200∆NSm/ZsG-infected mice at this time point, with one animal euthanized at 9 dpi (Panel X) showing markedly lower ZsG expression in the liver than the other 10200∆NSm/ZsG-infected mice euthanized at 7 and 9 dpi.

To investigate the potential differences in tissue distribution between 10200/ZsG and 10200∆NSm/ZsG, gross ZsG fluorescence was visualized in situ in infected Ifnar^-/-^ mice at the time of euthanasia (2 dpi, 4 dpi, and in terminal animals). At 2 dpi, fluorescence was not detected in any tissue in either the 10200/ZsG- or 10200∆NSm/ZsG-infected mice, with the exception of a focal signal observed unilaterally in a draining axillary lymph node of one 10200∆NSm/ZsG-infected mouse. These findings paralleled the absence or low levels of viral RNA detected by qRT-PCR at this time (Figure 4, Panels II and III). At 4 dpi, ZsG fluorescence (widely distributed but not uniform) could be visualized in the liver and multiple peripheral lymph nodes, including cervical and inguinal lymph nodes, of one of the three 10200/ZsG-infected mice (Figure 4, Panel IV). This detectable fluorescence was associated with higher viral RNA levels (4 × 10^7^–2 × 10^8^ copies/µL in the spleen, liver, and blood) in this animal compared to the other two 10200/ZsG-infected mice euthanized at 4 dpi, in which no fluorescence could be visualized. The liver with detectable fluorescence was paler on gross examination than livers without signal. Fluorescence was not detected in any of the three 10200∆NSm/ZsG-infected mice euthanized at this time point (Figure 4, Panel V). In terminal 10200/ZsG-infected mice euthanized due to clinical disease at either 5 or 6 dpi, fluorescent signal was strongest in the liver, where it was intense and uniformly distributed. ZsG fluorescent signal was also evident in the lymph nodes, spleen, kidney, gastrointestinal tract, and reproductive organs (Figure 4, Panels VI–VII). While widely disseminated, fluorescent signal was visible in terminal 10200∆NSm/ZsG-infected mice in the same organs as in 10200/ZsG-infected mice; the signal intensity varied among individuals (Figure 4 Panels VIII–X). This was particularly apparent in the liver, where pallor on gross examination correlated with ZsG intensity, and a markedly weaker ZsG signal was seen in one mouse euthanized at 9 dpi compared to mice euthanized at 7 and 9 dpi. All animals euthanized in the terminal 10200∆NSm/ZsG group displayed similar clinical signs (ruffled fur and moribund) and progressively lost 17%–19% of body weight compared to −1 dpi baselines in the 2 days prior to euthanasia.

### 3.4. Comparative Pathology of Mice Infected with 10200/ZsG or 10200∆NSm/ZsG

Histopathology and immunohistochemistry were performed on tissues from all mice to compare pathology in 10200∆NSm/ZsG-infected and 10200/ZsG-infected mice. Overall, presence of pathology and immunostaining correlated with the extent of gross tissue fluorescence in mice infected with 10200/ZsG or 10200∆NSm/ZsG at all time points and in all examined tissues (liver, spleen, lymph nodes, brain, eye, kidney, adrenal gland, lung, heart, and gastrointestinal tract). Overt tissue pathology was limited to the liver and spleen and other lymphoid tissues (peripheral lymph nodes and gut-associated lymphoid tissue) for both groups. Differences in pathology were most evident and distinctive in the liver. Both hepatic and splenic pathology and immunostaining were less pronounced and had a delayed onset in the 10200∆NSm/ZsG mice compared to the 10200/ZsG mice.

**Figure 5:**
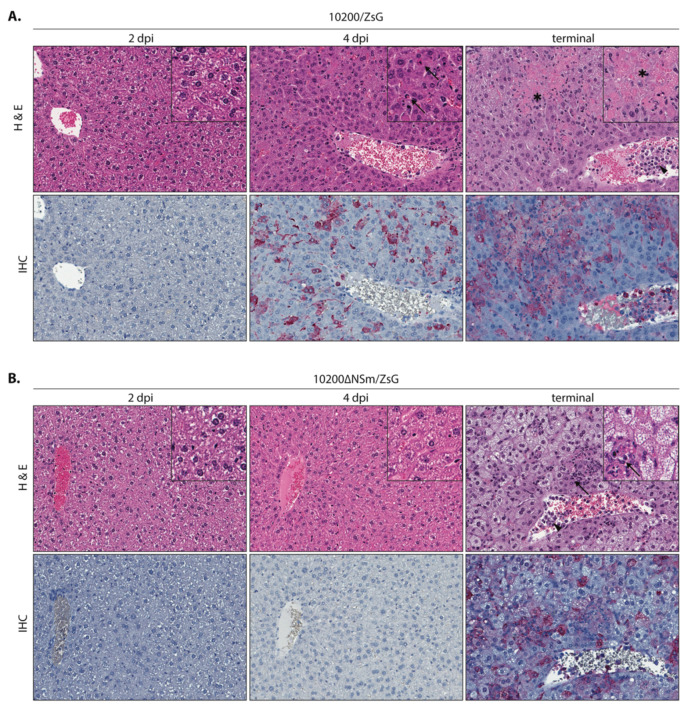
Liver pathology and CCHFV antigen distribution visualized by immunohistochemistry in Ifnar^−/−^ mice infected with 10200/ZsG or 10200ΔNSm/ZsG. (**A**) Mice infected with 10200/ZsG displayed no pathology or CCHFV immunostaining 2 dpi (left panel). At 4 dpi, 1 of 3 animals had scattered, mostly single-cell hepatocyte necrosis (arrow) with immunostaining (red) of hepatocytes and Kupffer cells (middle panel). All terminal animals (euthanized 5–6 dpi) had widespread regions of confluent hepatocyte necrosis (*) and immunostaining. Increased numbers of intravascular leukocytes (arrowhead) with immunostaining were also seen (right panel). (**B**) Histopathologic evidence of liver damage and immunostaining was delayed in 10200ΔNSm/ZsG-infected mice, with no pathology or CCHFV immunostaining detected 2 dpi or 4 dpi (left and middle panels). In terminal 10200ΔNSm/ZsG animals that succumbed to infection (7–9 dpi), extensive but sublethal hepatocyte injury was characterized by diffuse hepatocyte swelling and vacuolation and was accompanied by scattered necrosis and neutrophilic inflammation (arrows). Increased intravascular leukocytes (arrowhead) and immunohistochemical staining patterns (red) were similar to those seen in terminal 10200/ZsG animals. Top row: hematoxylin-eosin; original magnification: 200× (inset 400×). Bottom row: CCHFV immunoalkaline phosphatase staining with naphthol fast red substrate, hematoxylin counterstain; original magnification: 200×.

The livers of mice infected with 10200/ZsG (Figure 5a) showed no pathologic changes or viral immunostaining at 2 dpi. At 4 dpi, the single mouse showing gross tissue fluorescence had scattered single-cell and confluent hepatocyte necrosis with minimal acute inflammation in the liver and scattered lymphocytolysis with increased histiocytes in the spleen (Figure S2). Terminal 10200/ZsG animals had widespread, single-cell and confluent hepatocyte necrosis, still with minimal inflammation, and central veins frequently contained prominent leukocytes. The spleens had increased lymphocytolysis. Only tissues from animals with gross fluorescence and microscopic pathology (one animal euthanized 4 dpi and all terminal animals (n = 9)) had detectable CCHFV immunostaining. Immunostaining in the liver and lymphoid tissues correlated with the extent of histopathologic changes and was localized to necrotic and viable hepatocytes, Kupffer cells, rare endothelial cells, and intravascular leukocytes in the liver; to macrophages and apoptotic debris in splenic white pulp; and to histiocytes in red pulp. Rare, scattered staining was also variably seen in intravascular leukocytes and rare endothelial or interstitial cells in other tissues (brain, heart, lung, kidney, and adrenal gland).

The livers of mice infected with 10200ΔNSm/ZSG (Figure 5b) had no significant pathologic changes or immunostaining in any tissue at 2 dpi or 4 dpi. The livers of terminal 10200∆NSm/ZsG-infected mice showed diffuse, sublethal hepatocyte injury (swelling and vacuolation) with scattered foci of hepatocellular necrosis and neutrophilic inflammation. Leukocytosis was also seen. Lymphocytolysis and histiocyte infiltration in the spleen were similar but slightly less pronounced in the terminal 10200∆NSm/ZsG-infected mice than in 10200/ZsG-infected mice; the spleens of three of the four 10200∆NSm/ZsG-infected mice that succumbed to infection also displayed some degree of lymphoid expansion, including plasma cell proliferation (Figure S2). Very rare staining of intravascular, endothelial, or interstitial cells in other tissues was comparable to that seen in terminal 10200/ZsG animals. The mouse that survived 10200∆NSm/ZsG infection had rare, scattered, small foci of mixed inflammation without hepatocyte necrosis in the liver and only rare lymphocytolysis in the spleen; CCHFV immunostaining was negative in all tissues.

## 4. Discussion

Here, using reverse genetics, we successfully generated a CCHFV mutant virus lacking the coding region for NSm. The in vitro consequences of NSm deletion were mild, with moderately slower but non-significant differences in growth kinetics compared to wild-type. Furthermore, although challenge with the deletion mutant in Ifnar^-/-^ mice resulted in a mildly attenuated disease course and reduced lethality compared to the wild-type virus, the majority of mice succumbed to disease, supporting the ability of 10200∆NSm to result in lethal disease similarly to the wild-type virus.

Research to identify putative roles of NSm in the CCHFV replicative cycle has been limited to date, only recently becoming possible due to the establishment of the CCFHV reverse genetics system [15]. Although the removal of NSm did not significantly affect the viral growth kinetics in several cell lines, some phenotypic differences were noted. IFN-competent cells like A549, in general, do not support CCHFV growth as well as cells with dampened or deficient IFN responses [26]. Here, although not reaching statistical significance, the growth kinetics of both 10200∆NSm and 10200∆NSm/ZsG in A549 cells were slower compared to those of their respective NSm-expressing analogs, and the end point titers were lower. These differences were not observed in BSR-T7/5 and Huh7 cells, which are unable to mount a robust IFN response during CCHFV infection. Since NSm proteins have been reported to be non-essential in several other bunyaviruses, it was not unexpected that its removal had no significant in vitro consequences [8,9,10,11,12]. Furthermore, although the GPCs of all nairoviruses share a common structural arrangement in PreGn (mucin-like domain—GP38—Gn) and PreGc (ProGc, Gc), the majority of these GPCs do not encode an NSm homolog [7]. Indeed, only members of the Nairobi sheep disease nairovirus genogroup (Dugbe, Kupe, Hazara, Nairobi sheep disease, and CCHF viruses) encode a double-membrane-spanning NSm between the PreGn and PreGc [24]. Our data further emphasize that NSm is not essential for nairovirus replication in general, although its presence may increase viral fitness and perhaps contribute to pathogenicity in humans or to persistence in the tick reservoir.

In vitro studies can only provide limited insight into functional roles of viral proteins, and certain functions can only be revealed using in vivo models. Using the Ifnar^−/−^ mouse model, we further showed that NSm was not required for CCHFV replication or pathology in vivo and that the majority of infected mice still succumbed to disease. However, the clinical onset (e.g., weight loss and hypoactivity) and time to death were delayed. This pattern was supported by later ZsG fluorescence detection in situ on serial sampling, as well as more pronounced variation in the ZsG intensity and distribution in animals that succumbed to disease. Notably, in contrast to the 100% lethality well described in mice infected with wild-type CCHFV, one mouse (1 of 5) survived infection. Thus far, only RVFVΔNSm and SBVΔNSm have been tested in vivo [11,12]. Both mutant viruses demonstrated reduced virulence, suggesting that the delayed disease we observed in the 10200∆NSm/ZsG-infected mice may also be specific to the absence of NSm despite the comparable growth kinetics in vitro to wild-type virus. These data suggest that while NSm does not appear to be a prominent or essential virulence factor, it may contribute to pathogenesis. However, in contrast to studies on RVFV that were performed in immunocompetent models, studies of SBV and our study were limited to using IFN-deficient mice. If NSm has a role in disrupting the immune response to infection, these effects may have been dampened in vivo due to the nature of the experimental system used.

What role CCHFV NSm plays during infection is still unknown. Our findings and those of others strongly suggest the absence of a structural role for NSm and a lack of involvement in RNA replication. However, given the observation that similar domains are maintained in the viral genomes of several nairoviruses, it is likely that some positive selection pressure is at play. Roles for other bunyavirus non-structural M segment proteins have been elucidated and may suggest several exploratory avenues for further research. For example, RVFV NSm exerts an anti-apoptotic effect in mammalian cells by suppressing the activity of cellular caspases [27] and plays an important role in viral replication within the mosquito vector [28]. Research demonstrating strong positive selection for NSm in competent tick vectors may suggest that the latter is also true for CCHFV [29].

In summary, our data demonstrate that NSm is dispensable for CCHFV replication and pathogenesis, and do not support a critical role for CCHFV NSm in virus assembly, as reported for NSm proteins of some other viruses. However, the absence of NSm resulted in a protracted clinical course in infected Ifnar^−/−^ mice, suggesting NSm as a putative target for attenuation and therapeutic development. This knowledge may be used alone or in combination with other attenuation approaches to produce novel interventions for further evaluation both in vitro and in vivo.

## Supplementary Materials

The following are available online at https://www.mdpi.com/2076-2607/8/5/775/s1. Figure S1: Western blot analysis of recombinant virus protein expression. Relative levels of CCHFV nucleoprotein (NP), ZsGreen1 (ZsG), and CCHFV Gn and Gc glycoproteins expressed in A549, Huh7, or BSR-T7/5 cells infected at MOI 0.1 with either 10200, 10200/ZsG, or 10200∆NSm/ZsG. Cells were collected at 24, 48, and 72 h post infection (hpi). Tubulin was used as a loading control. Figure S2: Representative splenic pathology and CCHFV antigen detection by immunohistochemistry in terminal 10200/ZsG- and 10200ΔNSm/ZsG-infected mice. Lymphocytolysis with presence of apoptotic debris and prominent macrophages is more pronounced in terminal 10200/ZsG-infected mouse spleen (left panel) than in 10200ΔNSm/ZsG-infected mouse spleen (right panel). Three of four terminal 10200ΔNSm/ZsG that succumbed to infection also had evidence of lymphoid expansion, with plasma cell proliferation (*). In both groups, immunostaining (red) revealed CCHFV in macrophages and apoptotic cells. Top row: hematoxylin-eosin; original magnification 200×. Bottom row: CCHFV immunoalkaline phosphatase staining with naphthol fast red substrate, hematoxylin counterstain; original magnification 200×.
