# Supplementary figures and images for: The Crimean-Congo Hemorrhagic Fever Virus NSm Protein Is Dispensable for Growth In Vitro and Disease in Ifnar-/- Mice
Source: Microorganisms. 2020 May 21;8(5):775. doi: 10.3390/microorganisms8050775 (PMC7285326; doi:10.3390/microorganisms8050775)

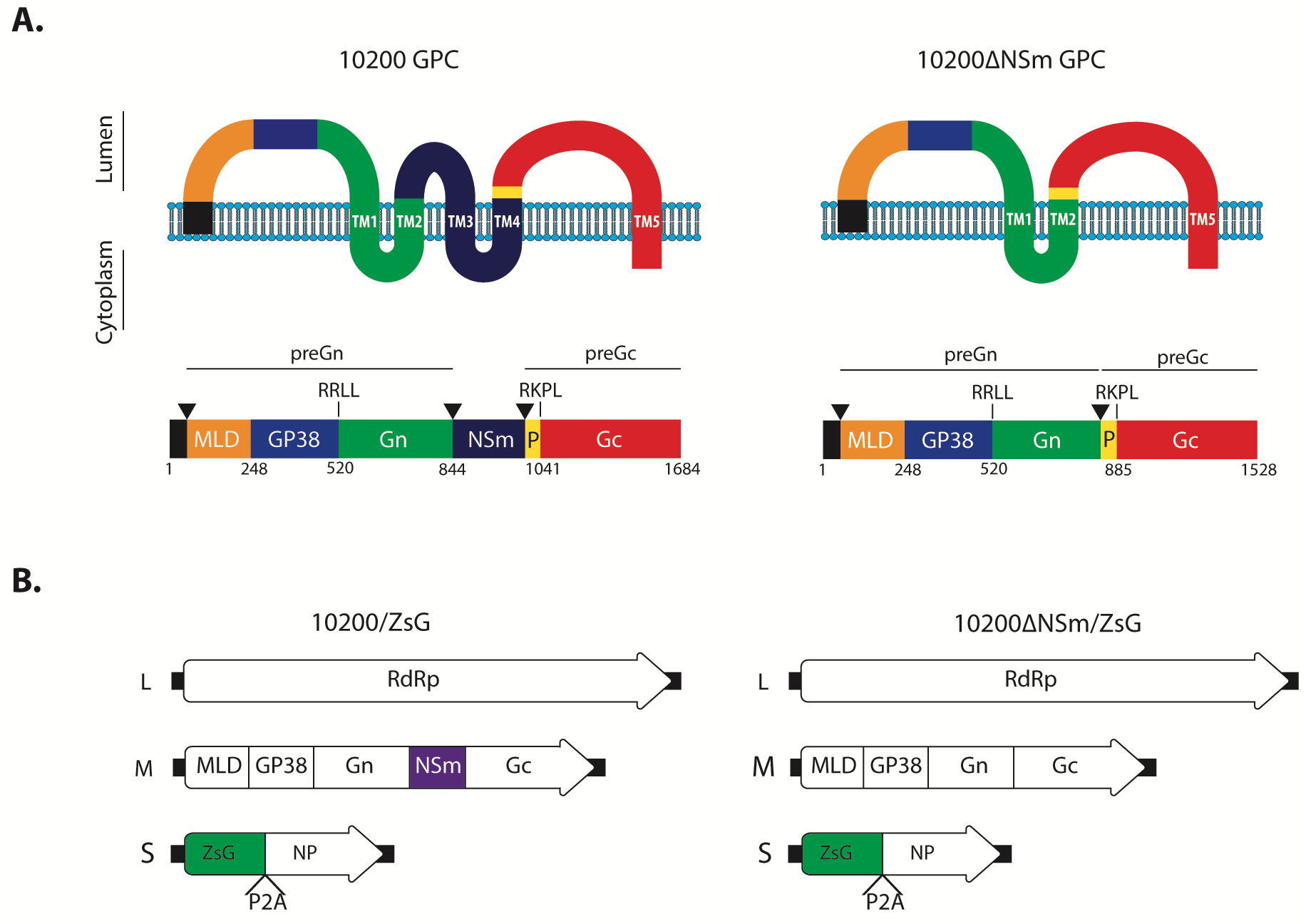

Supplement: Supplementary file 1 [file microorganisms-08-00775-s001.zip › Figure 1.tif]

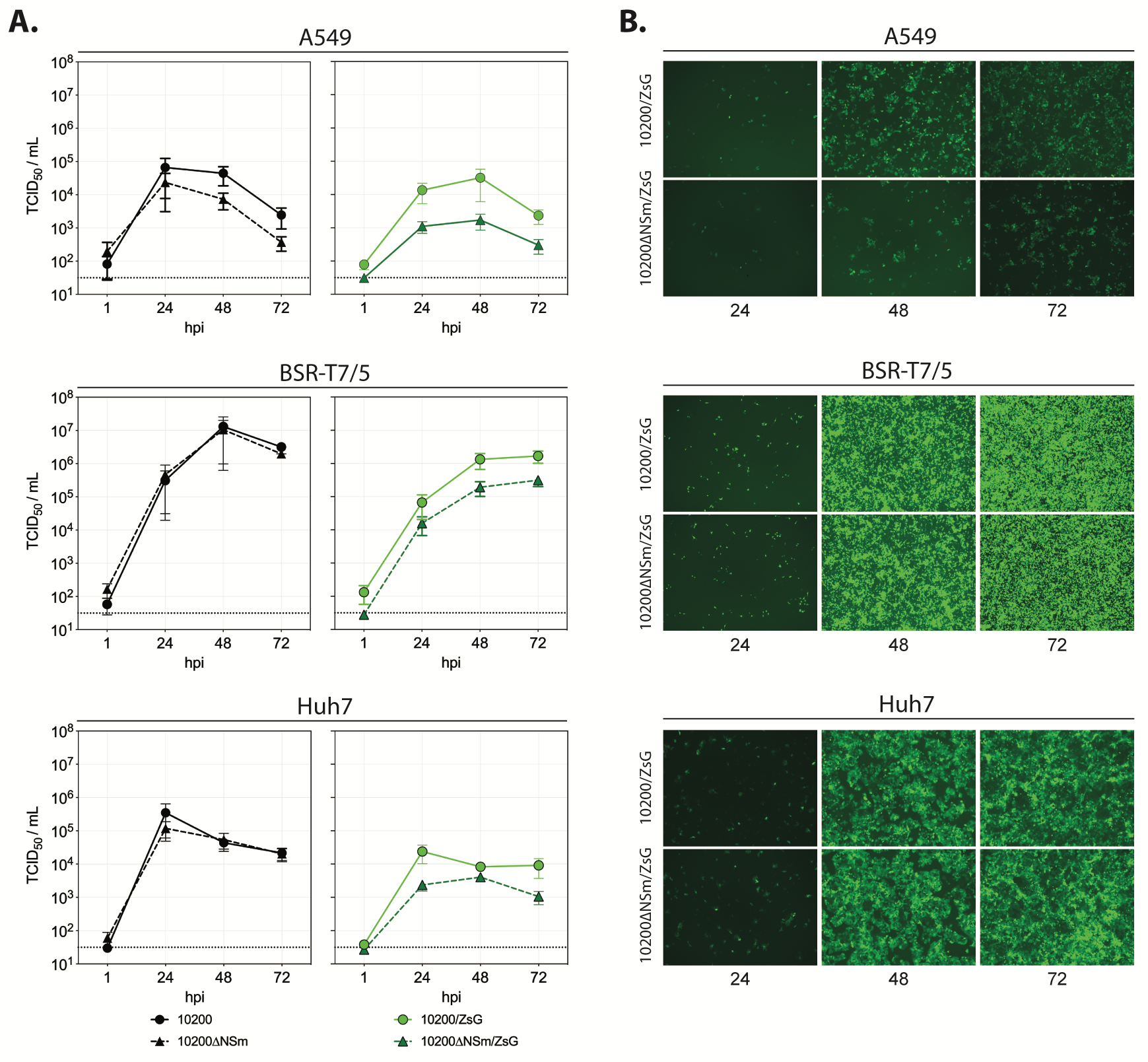

Supplement: Supplementary file 1 [file microorganisms-08-00775-s001.zip › Figure 2.tif]

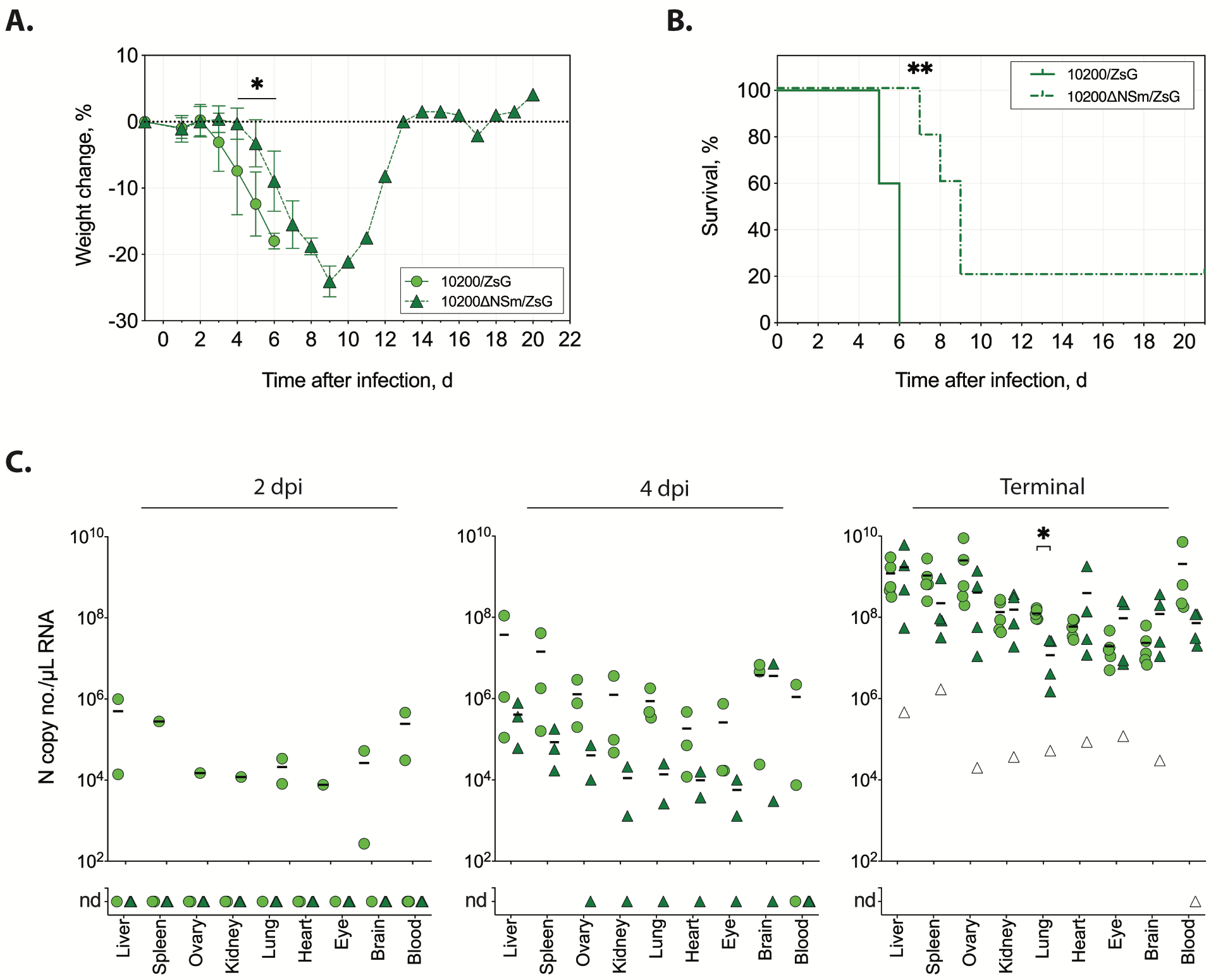

Supplement: Supplementary file 1 [file microorganisms-08-00775-s001.zip › Figure 3.tif]

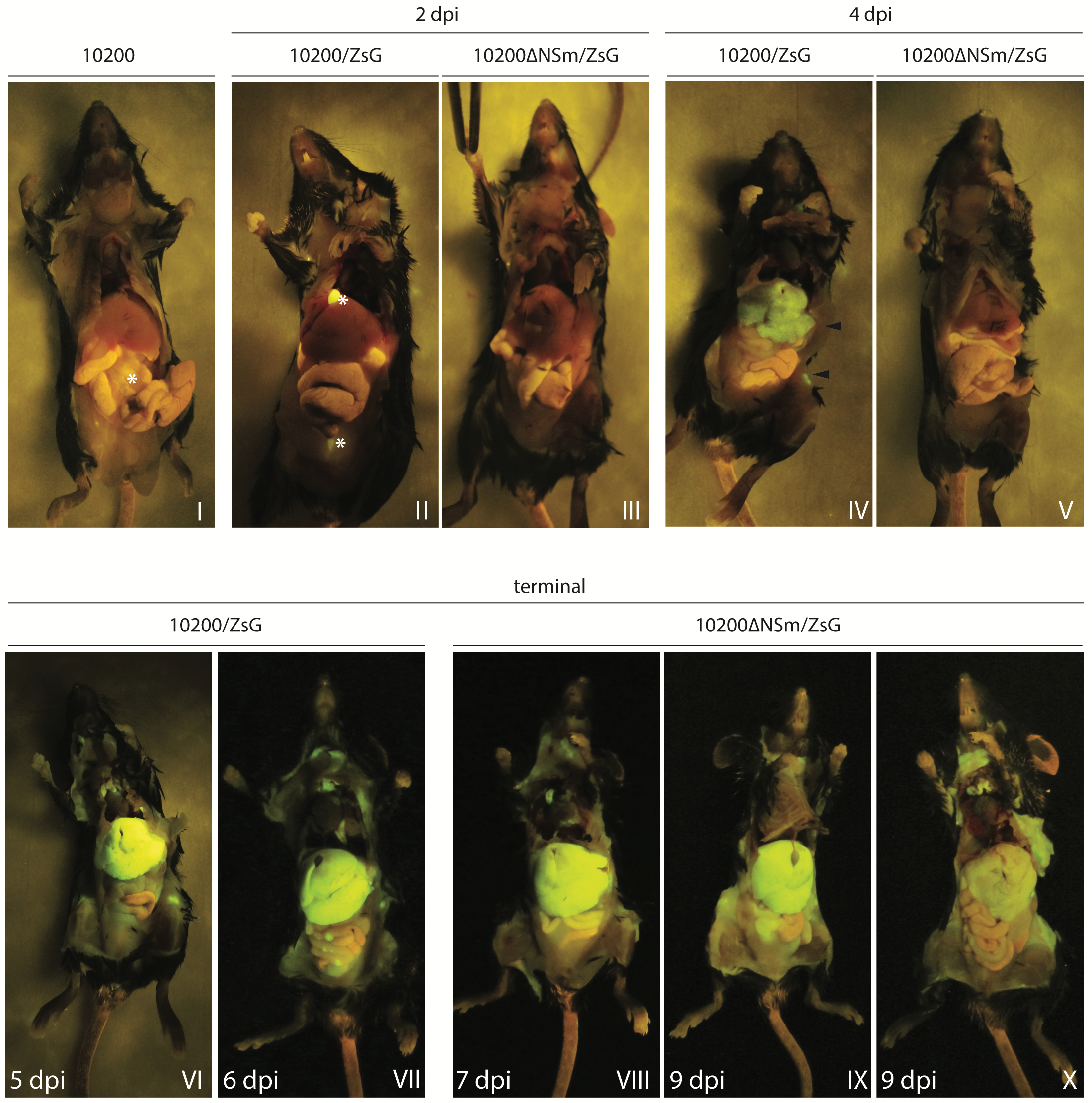

Supplement: Supplementary file 1 [file microorganisms-08-00775-s001.zip › Figure 4_corrected.tif]

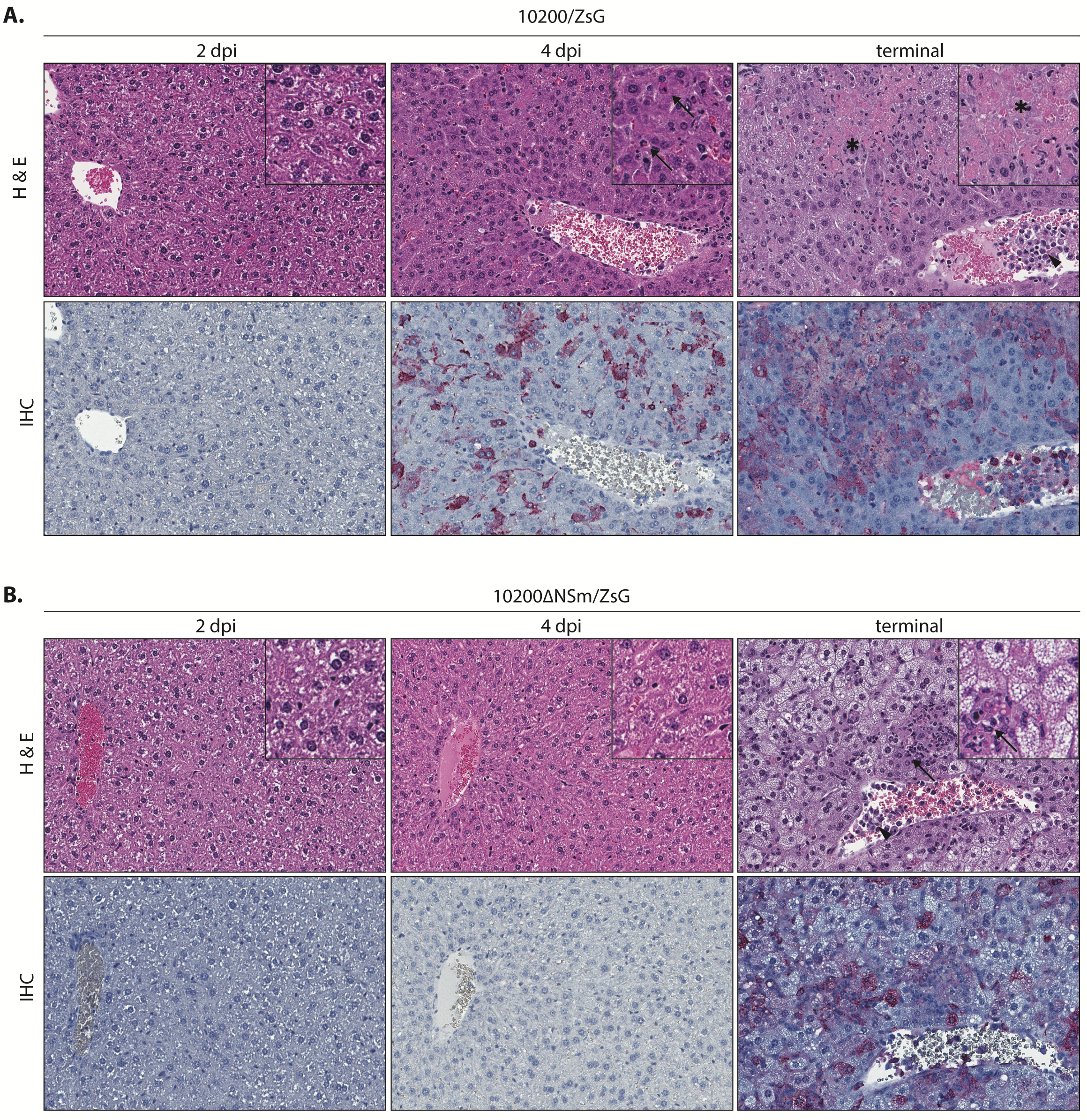

Supplement: Supplementary file 1 [file microorganisms-08-00775-s001.zip › Figure 5.tif]

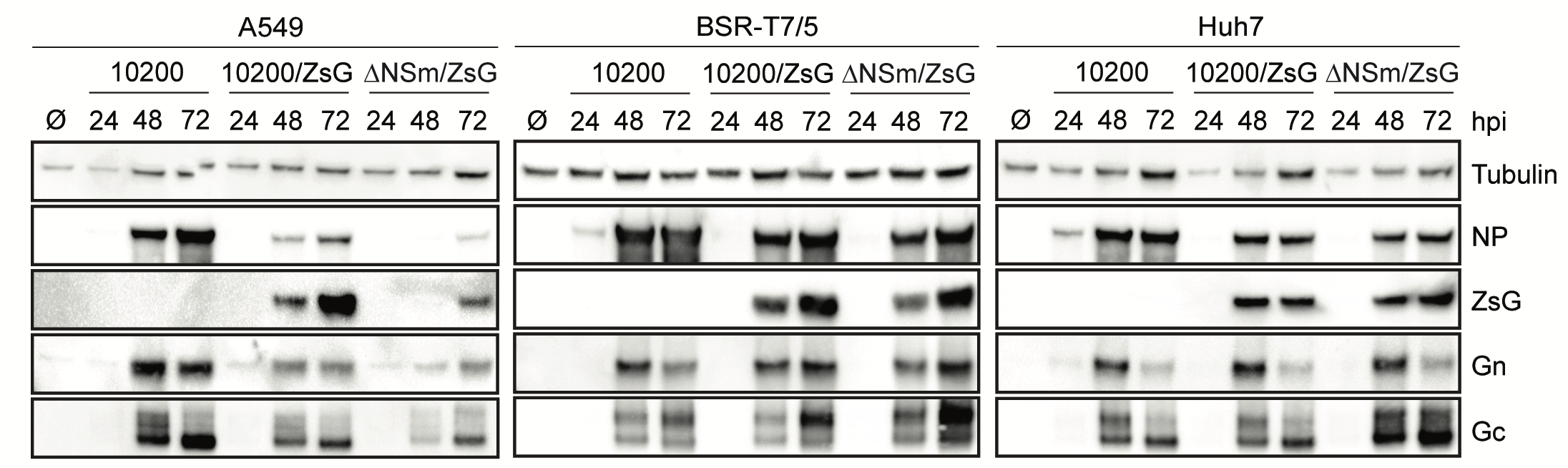

Supplement: Supplementary file 1 [file microorganisms-08-00775-s001.zip › Supplemental Figure 1.tif]

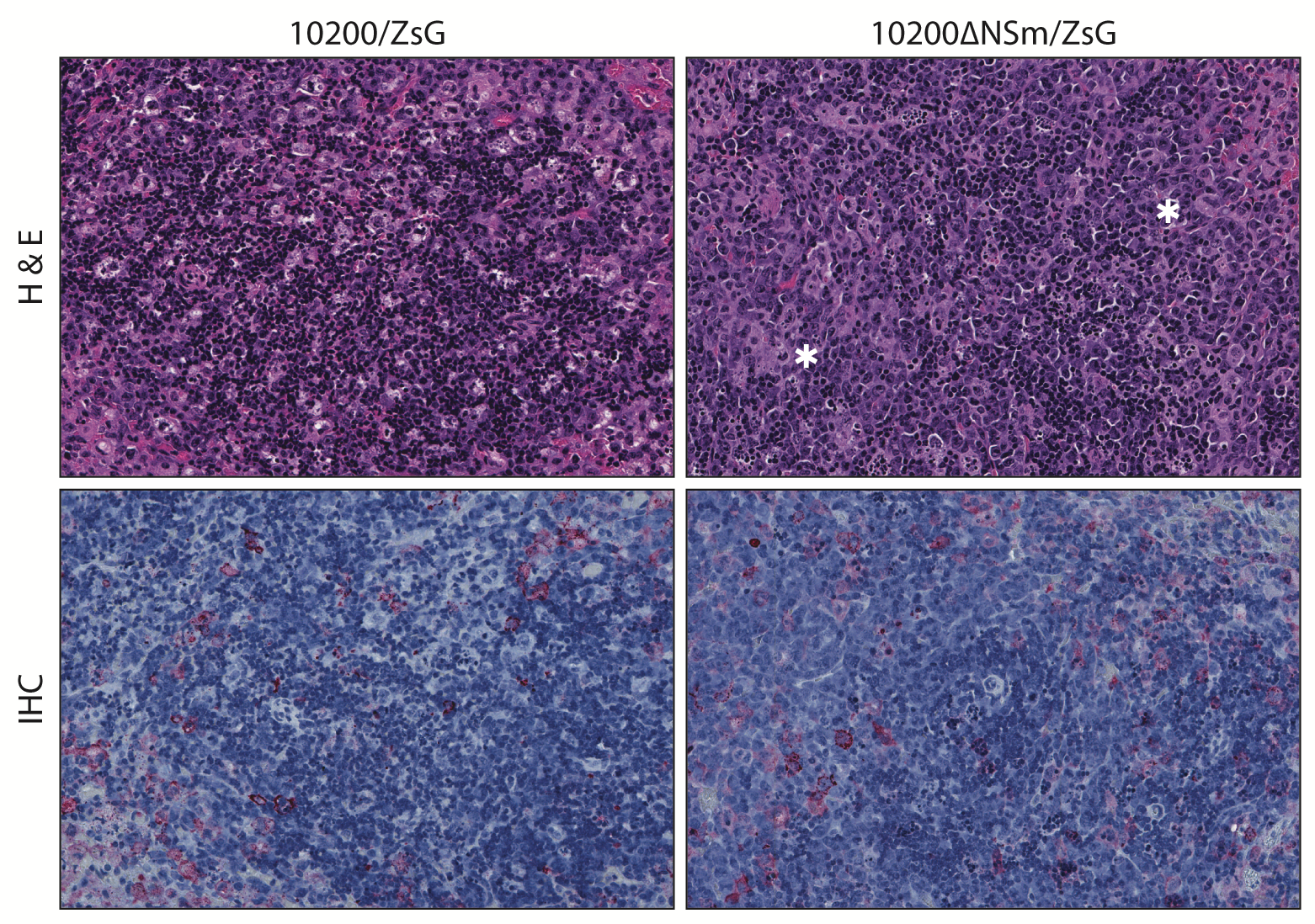

Supplement: Supplementary file 1 [file microorganisms-08-00775-s001.zip › Supplemental Figure 2.tif]
